# Climate trends and maize production nexus in Mississippi: empirical evidence from ARDL modelling

**DOI:** 10.1038/s41598-023-43528-6

**Published:** 2023-10-03

**Authors:** Ramandeep Kumar Sharma, Jagmandeep Dhillon, Pushp Kumar, Raju Bheemanahalli, Xiaofei Li, Michael S. Cox, Krishna N. Reddy

**Affiliations:** 1 Department of Plant and Soil Sciences, Mississippi State University, Mississippi, USA; 2 Department of Economics, Manipal University Jaipur, Dhami Kalan, Rajasthan India; 3 Department of Agricultural Economics, Mississippi State University, Mississippi, USA; 4 Crop Production Systems Research Unit, United States Department of Agriculture (USDA)-Agricultural Research Service (ARS), Stoneville, MS USA

**Keywords:** Plant physiology, Plant sciences, Climate sciences

## Abstract

Climate change poses a significant threat to agriculture. However, climatic trends and their impact on Mississippi (MS) maize (*Zea mays* L.) are unknown. The objectives were to: (i) analyze trends in climatic variables (1970 to 2020) using Mann–Kendall and Sen slope method, (ii) quantify the impact of climate change on maize yield in short and long run using the auto-regressive distributive lag (ARDL) model, and (iii) categorize the critical months for maize-climate link using Pearson’s correlation matrix. The climatic variables considered were maximum temperature (Tmax), minimum temperature (Tmin), diurnal temperature range (DTR), precipitation (PT), relative humidity (RH), and carbon emissions (CO_2_). The pre-analysis, post-analysis, and model robustness statistical tests were verified, and all conditions were met. A significant upward trend in Tmax (0.13 °C/decade), Tmin (0.27 °C/decade), and CO_2_ (5.1 units/decade), and a downward trend in DTR ( − 0.15 °C/decade) were noted. The PT and RH insignificantly increased by 4.32 mm and 0.11% per decade, respectively. The ARDL model explained 76.6% of the total variations in maize yield. Notably, the maize yield had a negative correlation with Tmax for June, and July, with PT in August, and with DTR for June, July, and August, whereas a positive correlation was noted with Tmin in June, July, and August. Overall, a unit change in Tmax reduced the maize yield by 7.39% and 26.33%, and a unit change in PT reduced it by 0.65% and 2.69% in the short and long run, respectively. However, a unit change in Tmin, and CO_2_ emissions increased maize yield by 20.68% and 0.63% in the long run with no short run effect. Overall, it is imperative to reassess the agronomic management strategies, developing and testing cultivars adaptable to the revealed climatic trend, with ability to withstand severe weather conditions in ensuring sustainable maize production.

## Introduction

Maize is the most important cereal, known as the “queen of cereals^1^.” The United States (US) is the leading producer, followed by China, Brazil, and Argentina^2^. The US contributes 32% to global production, and 60% of total production is exported^2^. Within the US, Mississippi (MS) is the state that contributes 748.3 million USD annually to national maize revenue^3^. Mississippi has 0.64 million acres under maize cultivation^4^. Mississippi has eight of the total twelve soil types, 60% of cropland is irrigated (by center pivot and furrow), and maize is grown on raised beds^5,6^. Mississippi has registered its maize yield progressing at a faster annual growth rate than the US for the past two decades^7^. As a result, MS actual maize yield surpassed the US in 2000; the current yields for MS and the US are 12.51 and 11.87 Mg ha^-1^, respectively^4^. Over the past half-century, MS has experienced a rapid increase (173%) in the harvested acres for maize compared to the US average (47%)^4^. More intriguingly, MS maize still has a considerable yield gap of 2 to 5.6 Mg ha^-1^, or 14 to 31%, at the state level when compared to the highest achievable yield under best management practices^7^. Closing these yield gaps is critical for economic benefits, reducing food prices, and consequently improving food security^8^. Strategies to close existing yield gaps via research necessitate a broader understanding of the causal factors and their extent on variations in crop yield^9^.

The factors that govern crop production and its variability include genetics, environment, and management such as soil properties, and agronomic management for instance fertilization, irrigation, tillage, planting dates, row-to-row width, planting population, planting time, depth, etc.,^10,11^. However, amongst all, the climate is noted to be the major uncontrollable contributor affecting crop production, with the proven potential to explain up to or even greater than 60% of the global crop yield variations^12^. Numerous studies on wheat (*Triticum aestivum* L*.*)^13–16^, maize^17–19^ and rice (*Oryza sativa* L*.*)^20,21^ has demonstrated a consensus on crop-climate link in cereals. Based on region-specific studies, the crop-climate association was found to be strong, ranging 22–60%, 40–71.3%, and 67–92% in wheat, maize, and rice, respectively. The same has been confirmed by global studies for other crops as well^22–25^. Specifically, in maize, Rizzo et al.^26^ attempted to separate climate, management, and genetic factors and deduced that climate change (48%) explained most of the yield variation, followed by management (39%), and genetics (13%). Given the alarming rate of future climate warming, almost 1.5 °C upsurge, precipitation (PT) irregularities (24–40%) combined with increased carbon emissions, the coefficient of yield dependability on climate is expected to rise further by 47% in 2050^27^.

Climatic trends induce biotic and abiotic stresses in plants by controlling microclimates around them, and influence evapotranspiration, gas exchange, resource use efficiency, plant-microbe relations, phenological processes, crop performance, and finally yield^28^. The severity of crop-climate links is determined by the magnitude and trend of change of climatic variables, which vary by region, and such estimates for MS are lacking^29^. Mississippi is in a climatically vulnerable southeastern region of the US, and has a significant agroeconomic impact^30,31^. Also, Mississippi agriculture relies on reduced capital investments and infrastructural inputs, removing several choices for combating climate-related negative consequences^32,33^. Even so, only a few climate-crop studies were conducted so far for MS^34–37^, and even fewer on maize^21,38,39^. Therefore, the current study is aimed at calculating (i) the trend in climatic variables, namely, daily maximum temperature (Tmax), daily minimum temperature (Tmin), diurnal temperature range (DTR), precipitation (PT), carbon emissions (CO_2_), and relative humidity (RH) in MS during 1970–2020, and (ii) impact of change in these variables on MS maize yield. The novelty of this study lies in investigating climatic variables other than just temperatures and PT, monthly investigations of trends in climatic variables, pinpointing crucial months impacting maize and employing econometric method for the first time to explore crop-climate link in MS.

## Methodology

**Figure 1:**
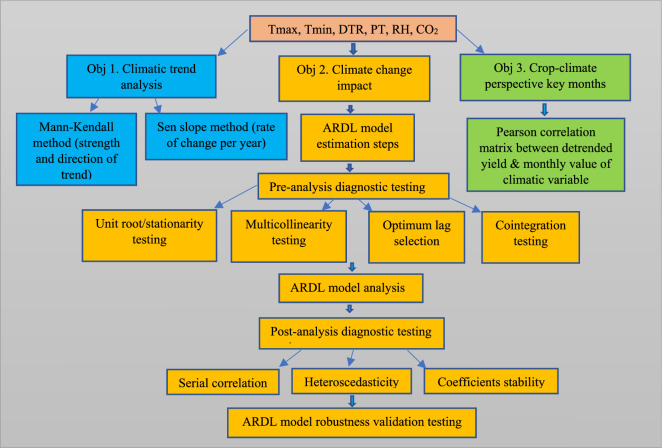
A step-by-step flowchart outlining the detailed methodology for the three different objectives. The first objective—estimating the trend for each of the six climatic variables—maximum temperature (Tmax), minimum temperature (Tmin), diurnal range (DTR), precipitation (PT), relative humidity (RH), and carbon dioxide emissions (CO_2_)—is shown in blue boxes on the left, the second objective—quantifying the overall impact of climatic variables on maize yield—are shown in yellow boxes in the middle, and the third objective workflow—identifying the key months for crop-climate linkage—are shown in green boxes on the right.

A detailed step-by-step outline of the various methodologies used to accomplish the study's objectives is displayed in Fig. 1. The sections below provide a detailed discussion on the various methodology components, including data, study model specifications, and the estimation procedures involved.

### Data

**Figure 2:**
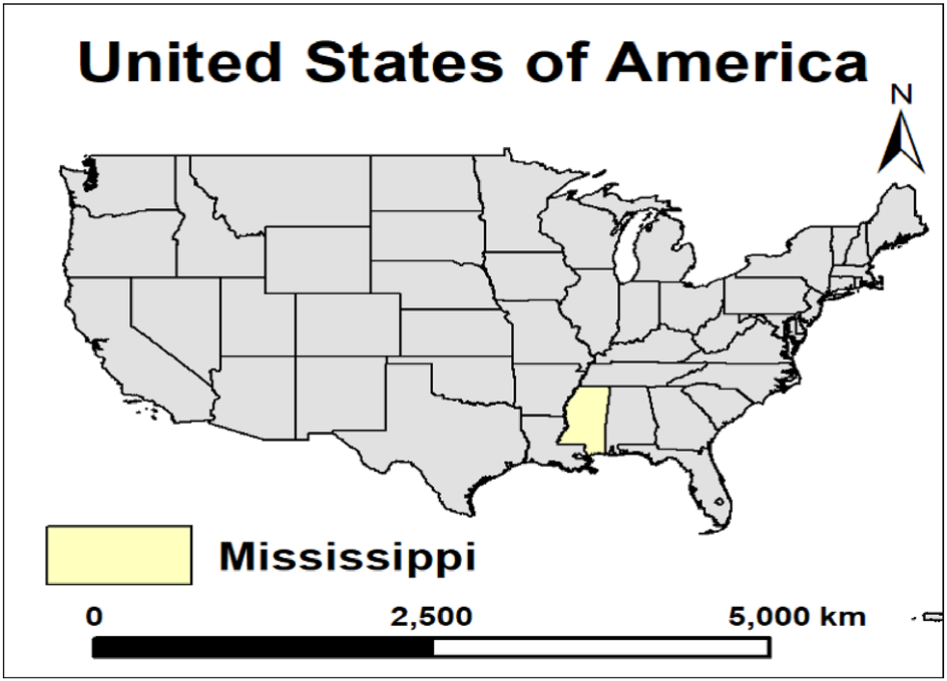
The study area (Mississippi state) highlighted on the USA map.

The present study utilized the past 50 years of time-series dataset for MS (Fig. 2), from 1970 to 2020 similarly to previous studies^12,40–42^.

As per World Meteorological Organization guidelines, 30 years (at minimum) dataset is recommended for climatic trend computations^43^. The response variable was maize yield, and the explanatory variables were Tmax, Tmin, DTR, PT, RH and CO_2_ (Fig. 1). Harvested area (HA) was included as an input control variable as suggested by Jan et al.^44^. Moreover, following Chandio et al.^40^, the Tmax, Tmin, DTR, and RH were averaged, and PT was totaled to maize growing season (MGS) for analyzing the impact of growing season anomalies. Also, the monthly averaged data of each variable was utilized to compute the month-wise climatic impact on maize. The MGS (March-September) was taken as per the USDA harvesting and planting dates handbook. The data on CO_2_ was available on a yearly average basis. The data were gathered from the USDA-NASS repository (https://www.nass.usda.gov/) for yield, National Oceanic and Atmospheric Administration (NOAA) database (https://www.noaa.gov/) for Tmax, Tmin, DTR, and PT, PRISM database (https://prism.oregonstate.edu/comparisons/) for RH, and US energy information administration (https://www.eia.gov/environment/emissions/state/) for CO_2_. There is a vast literature authenticating the use of time series data and the aforesaid data sources for crop-climate estimations^45–48^.

### Econometric model specification

The two-dimensional effects of climate change on crops include a short-term effect that is directly impacting the yield in the current and subsequent (residual effect) years^49,50^. This immediate effect accumulates to build the foundation for permanent effects, referred to as long-term effects, that ultimately influence the soil-forming processes, soil properties, microbial buildups in the soil, and nutrient-use abilities^51–53^. Therefore, the study evaluated both the short and long-term relationships between the variables using the widely used auto-regressive distributive lag (ARDL) bound-testing method^44,54–58^. The ARDL model is preferred over other statistical methods because it can efficiently run the analysis for both short-term and long-term relationships simultaneously at ceteris paribus keeping all other variables unchanged^55^. Moreover, the ARDL model accounts for previous year inputs/factors influencing the current year yield, by incorporating the “lag length” component in its functionality^59^. These factors could be residual effects of previous year fertilization especially if a granular form is applied, late season excessive rainfall, or maybe rollover effects of previous crop rotation^60,61^. By regressing the lag values of the regressors against the regressand, the lag length feature statistically advises the ARDL model on how far back in time it needs to go to capture the residual effect^62,63^. The ARDL model works well regardless of the integration level of the time series data *i.e.,* level (I = 0), at first difference (I = 1), or combination of I (0), and I (1)^56^. The ARDL approach is robust against endogeneity issues, which arises when the dependent variable tends to correlate with the error term in the regression model^64^, reducing residual correlation, and small sample sizes^54^. The ARDL has an intrinsic feature of error correction model (ECM) that estimates the pace (% per year) with which the short-term effects transfer cumulatively to form permanent basis for the long-term effects^54^. The following linear equation was used to evaluate short-term and long-term association of mentioned variables:

1 $$Y=f(Tmax, Tmin, DTR, Prec, RH, C{O}_{2}, HA )$$

The natural log form variables are suggested for time series data to smoothen multicollinearity and instability issues if any^56^.

2 $$ \begin{aligned} lnY_{t} = & \beta_{0} + \beta_{1} {\text{ln}}(Tmax)_{t} + \beta_{2} {\text{ln}}(Tmin)_{t} + \beta_{3} {\text{ln}}(DTR)_{t} + \beta_{4} {\text{ln}}(PT)_{t} \\ & + \beta_{5} {\text{ln}}(RH)_{t} + \beta_{6} {\text{ln}}(CO_{2} )_{t} + \beta_{7} {\text{ln}}(HA)_{t} + \varepsilon_{t} \\ \end{aligned} $$

where, ${Y}_{t}$ is maize yield (Mg ha^-1^) in year ***t***. Tmax, Tmin, and DTR are in (°C), PT in (mm), RH in (%), CO_2_ in metric ton, HA is maize harvested in hectares, ${\beta }_{0}$ is intercept, and ${\beta }_{1}, {\beta }_{2, }{ \beta }_{3, } {\beta }_{4}, {\beta }_{5}, {\beta }_{6}, {\beta }_{7}$ are coefficients of slopes in the function, and ${\varepsilon }_{t}$ is error term in time *t.*

#### Auto-regressive distributive lag (ARDL) bound test approach

The ARDL model equation adopted in similar previous studies^44,55,57^, is used here as follow:

3 $$ \begin{aligned} \Delta lnY_{it} = & \alpha_{0} + \mathop \sum \limits_{i = 1}^{n} \alpha_{1} \Delta {\text{ln}}(Y)_{t - i} + \mathop \sum \limits_{i = 1}^{n} \alpha_{2} \Delta ln\left( {Tmax} \right)_{t - i} \\ & + \mathop \sum \limits_{i = 1}^{n} \alpha_{3} \Delta ln\left( {Tmin} \right)_{t - i} + \mathop \sum \limits_{i = 1}^{n} \alpha_{4} \Delta ln\left( {DTR} \right)_{t - i} + \mathop \sum \limits_{i = 1}^{n} \alpha_{5} \Delta ln\left( {PT} \right)_{t - i} \\ & + \mathop \sum \limits_{i = 1}^{n} \alpha_{6} \Delta {\text{ln}}(CO_{2} )_{t - i} + \mathop \sum \limits_{i = 1}^{n} \alpha_{7} \Delta ln\left( {RH} \right)_{t - i} + \mathop \sum \limits_{i = 1}^{n} \alpha_{8} \Delta ln\left( {HA} \right)_{t - i} \\ & + \mathop \sum \limits_{i = 1}^{n} \gamma_{1} \Delta ln\left( Y \right)_{t - i} + \mathop \sum \limits_{i = 1}^{n} \gamma_{2} \Delta {\text{ln}}(Tmax)_{t - i} + \mathop \sum \limits_{i = 1}^{n} \gamma_{3} \Delta ln\left( {Tmin} \right)_{t - i} \\ & + \mathop \sum \limits_{i = 1}^{n} \gamma_{4} \Delta ln\left( {DTR} \right)_{t - i} + \mathop \sum \limits_{i = 1}^{n} \gamma_{5} \Delta ln\left( {PT} \right)_{t - i} + \mathop \sum \limits_{i = 1}^{n} \delta_{6} \Delta ln\left( {CO_{2} } \right)_{t - i} \\ & + \mathop \sum \limits_{i = 1}^{n} \gamma_{7} \Delta ln\left( {RH} \right)_{t - i} + \mathop \sum \limits_{i = 1}^{n} \gamma_{8} \Delta {\text{ln}}(HA)_{t - i} + \emptyset (ECT)_{t - i} + \varepsilon_{t} \\ \end{aligned} $$

where *Y* is maize yield, *t* is the time in year, *i* is the lag order with n is the highest lag value, ${\alpha }_{0}$ is the intercept, $\Delta$ denotes the first differencing, ${\varepsilon }_{t}$ is the error term, ${\alpha }_{1}$ to ${\alpha }_{8}$ represents coefficients of long term cointegration for different variables, ${\gamma }_{1}$ to ${\gamma }_{8}$ are short term coefficients for different variables, ECT is the error correction term and $\varnothing$ is its coefficient which determines the pace (% per year) by which short term climatic impacts cumulatively transfer to form basis for permanent long term effects.

The first differencing, as suggested in previous studies^23,65^, was applied as a technique to detrend the maize yield to account for the other yield impacting unobserved factors such as advancement in agricultural technology, progression of the adjustments in growers according to the management recommendations, and the infrastructural developments. The data on aforesaid factors was not available. Detrending is widely used in literature to exclude (minimize) the impact of such unobserved variables and to capture the sole impact of climate variables on crop yields^23,65^.

### Climatic trend analysis

The Mann-Kendall test^66,67^ and Sen slope method^68^ were employed to time series (1970–2020) data for all study variables to establish the trend on both monthly and growing seasonal timescale (Mar-Sep). Both these non-parametric tests are recommended by the World Meteorological Organization for climatic trend estimation^69^. The Kendall tau computes the direction and strength of the trend where positive sign of the coefficient indicates increasing (upward), negative sign signifies decreasing (downward) trend, and the magnitude of 0–0.25 (weak), 0.26–0.50 (fair), 0.51–0.75 (moderate), and values above 0.76 (strong) signifies the strength of the trend^70–72^. However, the Sen slope coefficient indicates the rate of change per year. For more detailed understanding on methodology of both these tests, readers are suggested to read Gocic and Trajkovic^73^ or Gujree et al.^74^ procedures.

### Estimation procedures

#### Unit tests

Units root problem arise when the mean, variances, and co-variances are time dependent or non-constant during the study timeframe^75^. Usually, unit root problems (non-stationarity) exist with time series data, if it exists, can cause spurious regression^76^. When a single coefficient fails to accurately reflect the true relationship between the study variables, false regression occurs, and the conclusions drawn may be untrue^76^. Hence, the Augmented Dickey-Fuller (ADF)^77^ and the Phillips–Perron tests (PP)^78^ unit root tests were performed. The results revealed that all the variables were stationary at level or first differencing, fulfilling the assumption of ARDL bound testing model (Table 1A).

**Table 1:** Pre-analysis diagnostic testing.

Variables	ADF		PP		
	Level	First difference	Level	First difference	
(A) Unit root test results following Augmented Dickey-Fuller (ADF) and Phillips-Perron (PP) tests of variables including maximum temperature (Tmax), minimum temperature (Tmin), carbon dioxide emission (CO_2_), harvested area (HA), precipitation (PT), and maize grain yield (Y)					
Tmax	− 6.276***		− 10.036***		
Tmin	− 6.340***		− 10.580***		
CO_2_	− 2.256	− 8.400***	− 2.264	− 8.357***	
HA	− 3.237	− 8.323***	− 3.170	− 10.284***	
PT	− 6.317***		− 6.287***		
Y	− 7.058***		− 7.054***		

Variable	Variance inflation factor (VIF)	Tolerance value (TOV)			
(B) Multicollinearity test results based on variance inflation factor (VIF) and tolerance value (TOV) tests of variables including maximum temperature (Tmax), minimum temperature (Tmin), carbon dioxide emission (CO_2_), harvested area (HA), and precipitation (PT)					
Tmax	4.512	0.221			
Tmin	4.126	0.242			
CO_2_	3.207	0.312			
PT	2.475	0.404			
HA	2.937	0.340			
Mean value	3.451	0.304			

Lag	SMLR	FPE	AIC	SIC	HQ
(C) Model’s lag selection criterion using sequential modified statistics test (SMLR), final prediction error (FPE) test, Akaike information criterion (AIC) method, Schwarz information criterion (SIC) method, and Hannan-Quinn information criterion (HQ) method					
0	NA	8.36e-13	− 10.783	− 10.544	− 10.693
1	177.455	4.28e-14	− 13.768	− 12.099*	− 13.142*
2	37.853	7.06e-14	− 13.350	− 10.249	− 12.188
3	26.476*	3.42e-14*	− 14.295*	− 9.7631	− 12.597
4	67.775	7.43e-14	− 13.990	− 8.0276	− 11.756

Test Statistic	Value	Significance (%)	Level I (0)	First difference I (1)	
(D) The ARDL bounds cointegration test results					
F-statistic	7.228	10	2.08	3	
		5	2.39	3.38	
		1	3.06	4.15	

“***”shows the significance level at 1%.

*Indicates lag order selected by the criterion, SMLR: sequential modified likelihood ratio test statistic, FPE: Final prediction error, AIC: Akaike information criterion, SC: Schwarz information criterion, HQ: Hannan-Quinn information criterion, and each test at 5% level of significance.

#### Multicollinearity testing

Analyses involving multiple variables may be susceptible to multicollinearity due to the propensity of variables to become correlated with one another^79^. To avoid overfitting in a regression model caused by multicollinearity, either the variables exhibiting it should be eliminated, or it needs to be verified that the data is free of multicollinearity, using tests such as the variance inflation factor (VIF) test and tolerance test^80^. The present study performed both these tests and found that the VIF value (3.45) and tolerance value (0.30) were within the permissible limits (Table 1B); VIF < 10 and tolerance value (TOV) > 0.1^42,79,80^, confirmed that multicollinearity was not an issue with the dataset (Table 1B).

#### Optimum lag selection

The ARDL model can determine the number of prior years to include in the model for regressing the explanatory variables (including their lag values) against the regressand (current year yield) by using the optimal lag number, to incorporate the previous years’ residual effects on current year maize yield^55^. The study used statistical tests such as Sequential modified likelihood ratio (SMLR) test, final prediction error (FPE) test, Akaike information criterion (AIC) method, Schwarz information criterion (SIC) method, and Hannan-Quinn information criterion (HQ) method, as guided by Agbenyo et al.^57^, and Warsame et al.^55^, to select optimum lag length for the model.

The appropriate lag length for the ARDL model was determined to be three (Table 1C), based on the minimum value generated by majority of the tests (SMLR, FPE, and AIC) utilized. The lag length of three signifies that the previous three years data needs to be considered to regress against the regressand for capturing residual effects.

#### Cointegration testing

The Wald F-test was used for the null and alternative hypotheses testing after running a regression to check for the existence of cointegration between regressors and regressand^44^. The two types of threshold values were produced, the upper bound threshold values were termed I (1), and the lower bound threshold values were termed I (0). The null hypothesis is accepted if the Wald F-statistics value is less than the lower bound (at I = 0) threshold value, indicating no relationship present between the regressand and regressors^41^. However, the null hypothesis is rejected if the Wald F-statistics value is higher than the upper bound (at I = 1) threshold value, indicating the presence of a relationship between the regressand and regressors^41^. The Wald F-test value (Table 1D) was estimated as 7.228, which, at the 1% significance level, was higher than the upper critical limit (4.15). The absence of cointegration was thus ruled out as the null hypothesis, and the presence of cointegration was determined at a 1% level of significance.

#### Post analysis diagnostic tests, and sensitivity/robustness check of ARDL model

After the ARDL model estimation, the study performed Breusch-Godfrey LM test (for serial correlation check), Breusch–Pagan–Godfrey test (for heteroscedasticity check), and cumulative sum (CUSUM) and cumulative sum of squares (CUSUMSQ) of recursive residuals tests (for stability check of the model coefficients), as suggested by the previous studies^58^.

**Figure 3:**
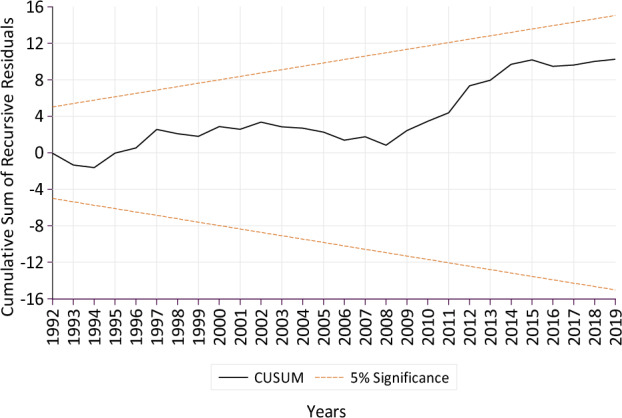
Cumulative sum (CUSUM) plot of recursive residuals of ARDL model with 95% confidence interval around the null.

**Figure 4:**
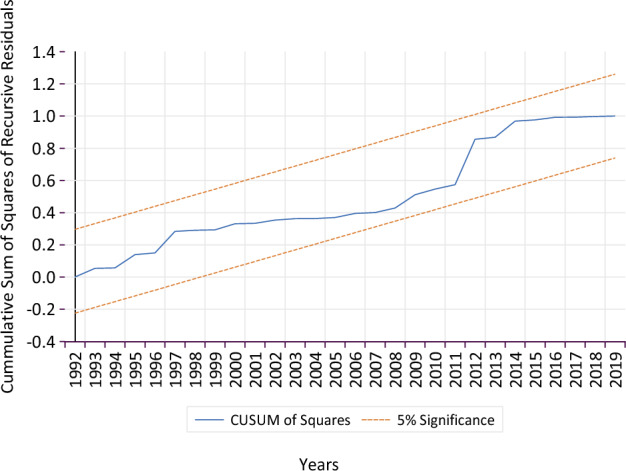
Cumulative sum (CUSUM) of squares Plot for recursive residuals of ARDL model with 95% confidence interval around the null.

The results confirmed that the functional model was free from serial correlation and heteroskedasticity (misspecifications) issues (Table 2A). The CUSUM and CUSUMSQ test graphs found that the parameter plot lines were consistent, stable, and stayed within critical bounds at the 5% level of significance (Figs. 3 and 4). Hence, confirming the accuracy and stability of short and long run model coefficients that affected the MS maize yield from 1970 to 2020. The CUSUM test can identify systematic, whereas the CUSUMSQ test identifies rapid and drastic variations from the constancy of the model coefficients^81^.

**Table 2:** Post analysis diagnostic testing.

Test	Statistics	Probability		
(A) Diagnostic test results following Breusch–Pagan–Godfrey test, Breusch-Godfrey LM test, cumulative sum (CUSUM) and cumulative sum of squares (CUSUMSQ) of recursive residuals tests, for the error terms of the regression equation obtained based on the ARDL model output				
BPG test for Heteroskedasticity	0.532	0.919		
BG LM test for Serial Correlation	0.841	0.443		
CUSUM	Stable	Figure 3		
CUSUM Squares	Stable	Figure 4		

Variable	Coefficient	Std. error	t-Statistic	Prob
(B) Results of fully modified ordinary least square (FMOLS) model for confirming the robustness and validation of the study model				
Tmax	− 14.133	4.073	− 3.469***	0.001
Tmin	7.735	2.524	3.064***	0.004
CO_2_	1.374	0.574	2.396**	0.021
HA	0.252	0.115	2.180**	0.035
PT	− 1.253	0.438	− 2.858***	0.007
C	26.614	10.959	2.429**	0.019
R-square	0.828			
Adjusted R-square	0.808			

Tmax represents maximum temperature, Tmin: minimum temperature, CO_2_: carbon emissions, HA: harvested acres for maize, and PT: precipitation.

After confirming the ARDL model's goodness of fit and predictive effectiveness by running post-analysis diagnostic tests, the sensitivity analysis was carried out using the fully modified ordinary least square (FMOLS) model to examine the robustness of the ARDL model functionality in long run. The FMOLS model showed that Tmax and PT had a negative impact on maize yield while Tmin and CO_2_ had a positive impact (Table 2B). These results are consistent with the long-run coefficients of the ARDL model, further validating the robustness of the model recommendations.

### Pearson’s coefficient of correlation matrix

Pearson’s coefficient of correlation between detrended (first differenced) yield and monthly averaged value of each climatic variable, as suggested by Eck et al.^82^, was calculated. Based on the strength of correlation, the months that had the greatest impact on maize yield were pinpointed.

## Results and discussions

The final regression fit equation used by the ARDL was a reduced model, which excluded DTR and RH since they were found to be non-significant and reducing the overall predictive efficiency of the model. Hence, the pre and post diagnostic tests (Tables 1, 2)—all of which were based on the ARDL model's assumptions—were only carried out for the variables that were part of the ARDL model. However, all variables were included for climatic trend analysis, and for calculating the Pearson’s correlation between detrended (first differenced) yield and monthly averaged values of climatic variables (Tables 3 and 4B).

**Table 3:** The summarized results of the Mann–Kendall test and the Sen slope method for trend estimation of variables including maximum temperature (Tmax), minimum temperature (Tmin), diurnal temperature range (DTR), precipitation (PT), relative humidity (RH), and carbon dioxide emission (CO_2_) in Mississippi from 1970 to 2020.

Series\test	Tmax		Tmin		DTR		PT		RH		CO_2_	
	Kendall tau	Sen slope	Kendall tau	Sen slope	Kendall tau	Sen slope	Kendall tau	Sen slope	Kendall tau	Sen slope	Kendall tau	Sen slope
March	0.139	0.032	0.146	0.030	0.012	0.001	− 0.095	− 0.193	0.047	0.021	–	–
April	0.014	0.003	0.101	0.015	− 0.078	− 0.008	0.090	0.194	0.157	0.060	–	–
May	0.103	0.012	0.178	0.022	− 0.092	− 0.009	− 0.087	− 0.183	0.003	0.000	–	–
June	0.051*	0.007*	0.373***	0.035***	** − **0.261**	** − **0.028**	0.095	0.163	0.125	0.036	–	–
July	− 0.006	− 0.001	0.262**	0.024**	** − **0.401***	** − **0.031***	0.119	0.147	0.068	0.022	–	–
August	0.066*	0.009*	0.299**	0.027**	** − **0.201*	** − **0.019*	0.158	0.269	− 0.009	− 0.004	–	–
September	0.143	0.021	0.183	0.027	0.006	0.001	− 0.063	− 0.112	− 0.110	− 0.060	–	–
MGS	0.176*	0.013*	0.422***	0.027***	** − **0.252**	** − **0.015**	0.057	0.432	0.027	0.011	0.669***	0.514***
Mean	28.56 °C		16.02 °C		12.54 °C		48.49 mm		66.73%		53.58 million metric tons (Mmt)	

Kendall tau negative (–) value signifies downward (decreasing) trend, and positive ( +) value indicates upward (increasing) trend with its value ranging between -1 and 1, and its absolute value signifies the strength of the trend. As the absolute value of Kendall tau approach 1, the strength of the trend becomes strong. The Sen slope value represents the rate of change (of variable) per year. Kendall tau is a pure number (unitless) as it is a correlation coefficient and Sen slope units are °C/year (for Tmax, Tmin, and DTR), mm/year (for PT), percentage/year (for RH), and Mmt/year (for CO_2_). The negative (–) value of Sen slope means the rate of decrease per year while the positive ( +) value represents the rate of increase per year. Significance: “*” *p* < 0.05, “**” *p* < 0.01, and “***” *p* < 0.001.

**Table 4:** Impact of climate change on maize yield.

Variable	Coefficient	Std. Error	t-Statistic	Prob	
(A) Calculated ARDL model estimates for short and long run effects of Tmax, Tmin, CO_2_, HA, and PT on maize yield (dependent variable)					
ARDL model long run effects					
Tmax	− 26.330	9.169	− 2.872***	0.008	
Tmin	20.684	6.731	3.073***	0.005	
CO_2_	0.629	0.976	0.644**	0.032	
HA	0.155	0.154	1.007	0.323	
PT	− 2.696	0.983	− 2.742**	0.011	
ARDL model short run effects					
Tmax	− 7.392	2.074	− 3.563***	0.001	
Tmin	2.361	1.340	1.760	0.091	
CO_2_	− 0.061	0.623	-0.098	0.922	
HA	0.018	0.093	0.198	0.844	
PT	− 0.645	0.249	− 2.587**	0.016	
C	44.329	25.660	1.728**	0.096	
ECM	− 0.302	0.038	− 7.892***	0.000	
R square	0.834				
Adjusted R square	0.766				

Growing season months	Climatic variables				
	Tmax	Tmin	DTR	PT	RH
(B) Pearson’s correlation matrix between the first differenced (detrended) yield and climatic variables (Tmax, Tmin, DTR, PT, RH) based on each month of MGS					
March	0.248	0.228	0.013	− 0.251	0.103
April	0.062	0.129	− 0.107	0.024	0.248
May	0.173	0.240	− 0.123	− 0.143	− 0.024
June	− 0.001**	0.485***	− 0.420**	0.267	0.226
July	− 0.159***	0.314*	− 0.472***	0.132	0.190
August	− 0.000	0.354**	− 0.319*	− 0.323*	0.022
September	0.213	0.231	− 0.019	− 0.098	− 0.126

“*” *p* < 0.05, “**” *p* < 0.01, and “***” *p* < 0.001.

Tmax represents maximum temperature, Tmin: minimum temperature, DTR: diurnal temperature range, CO_2_: carbon emissions, HA: harvested acres for maize, PT: precipitation, and ECM: error correction model. Significance codes: “*” *p* < 0.05, “**” *p* < 0.01, and “***” *p* < 0.001.

### Climatic trend analysis

**Figure 5:**
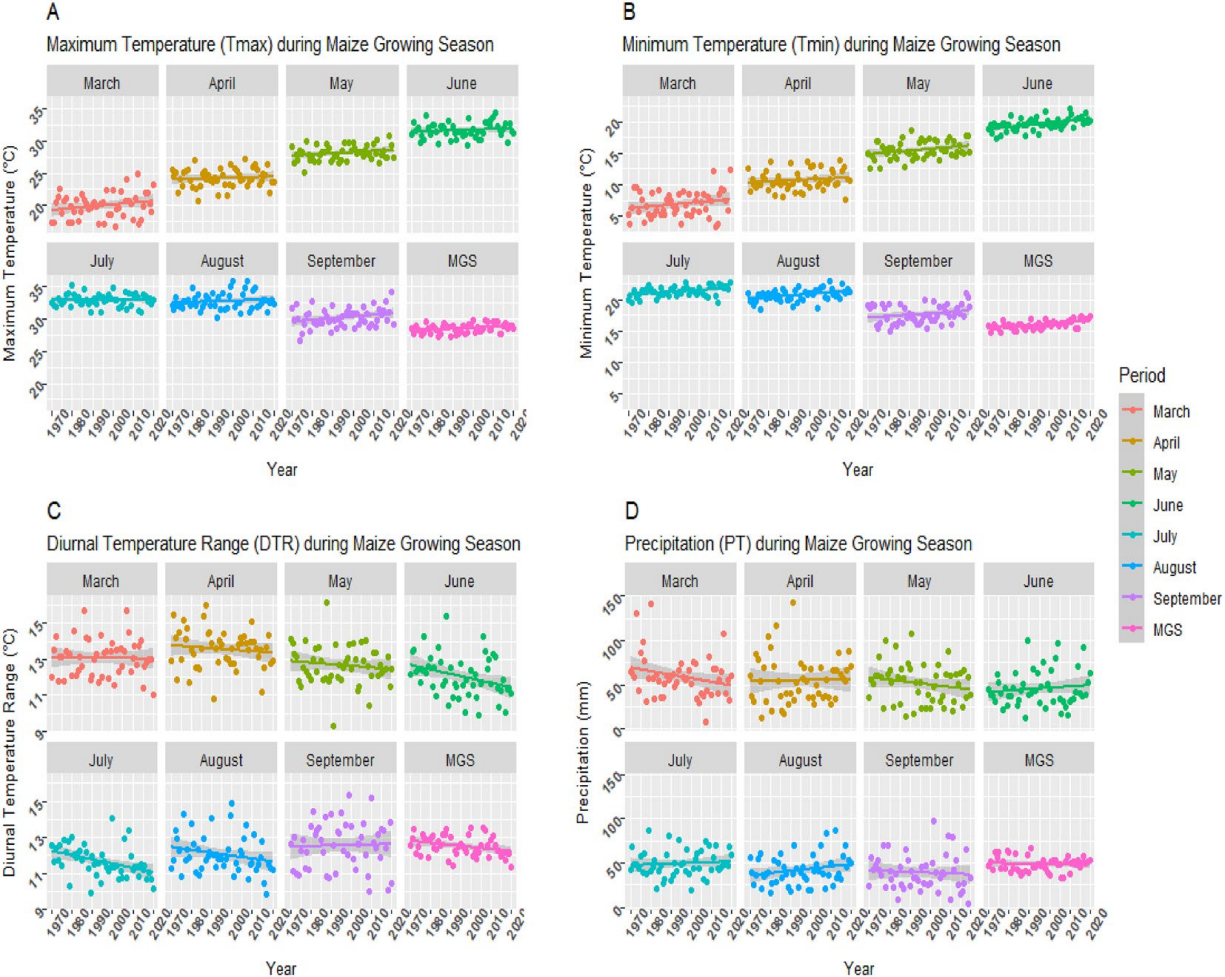
Trend lines for Tmax (**A**), Tmin (**B**), DTR (**C**), and precipitation (**D**) for maize growing season (MGS) and its individual months from 1970 to 2020 in Mississippi. Each figure is faceted by months from March to September and average of all months all together in MGS.

Tmax increased by 0.13 °C per decade in MGS, while Tmin increased by 0.27 °C per decade, which is 107.67% faster than Tmax (Table 3). Other studies have found similar unsymmetric Tmin-Tmax warming rates^83–86^. There was an upward trend for Tmax for MGS, specifically for June and August, but it was weak, as magnitude of correlation strength was less than 0.25 (Fig. 5A; Table 3). July was the only month that experienced a Tmax decreasing trend (Fig. 5A), yet non-significant (Table 3).

In contrast, MGS shows an upward trend for Tmin, increasing by 0.27 °C per decade in the last five decades (Fig. 5B; Table 3). Tmin warming rates ranged between 0.24 and 0.35 °C per decade in June, July, and August of MGS (Table 3). June, Tmin had the greatest rise, adding 0.35 °C per decade to global warming (Table 3). The equivalent rising trends were seen by Eck et al.^82^ and Sharma et al.^87^ in MGSs in the southeastern part of the US.

In recent years, the DTR (Tmax-Tmin) has been recognized as another climatic variable that is essential for diagnosis, particularly under rising unsymmetrical warming scenarios^88,89^. There was a downward trend for DTR in June, July, and MGS, and a weak trend for August (Fig. 5C). In MGS, the DTR decreased by 0.15 °C per decade, but in June, July, and August, it decreased by 0.19–0.31 °C per decade (Table 3). These rates are comparable with the computations of Sun et al.^90^ for the other maize-growing regions.

**Figure 6:**
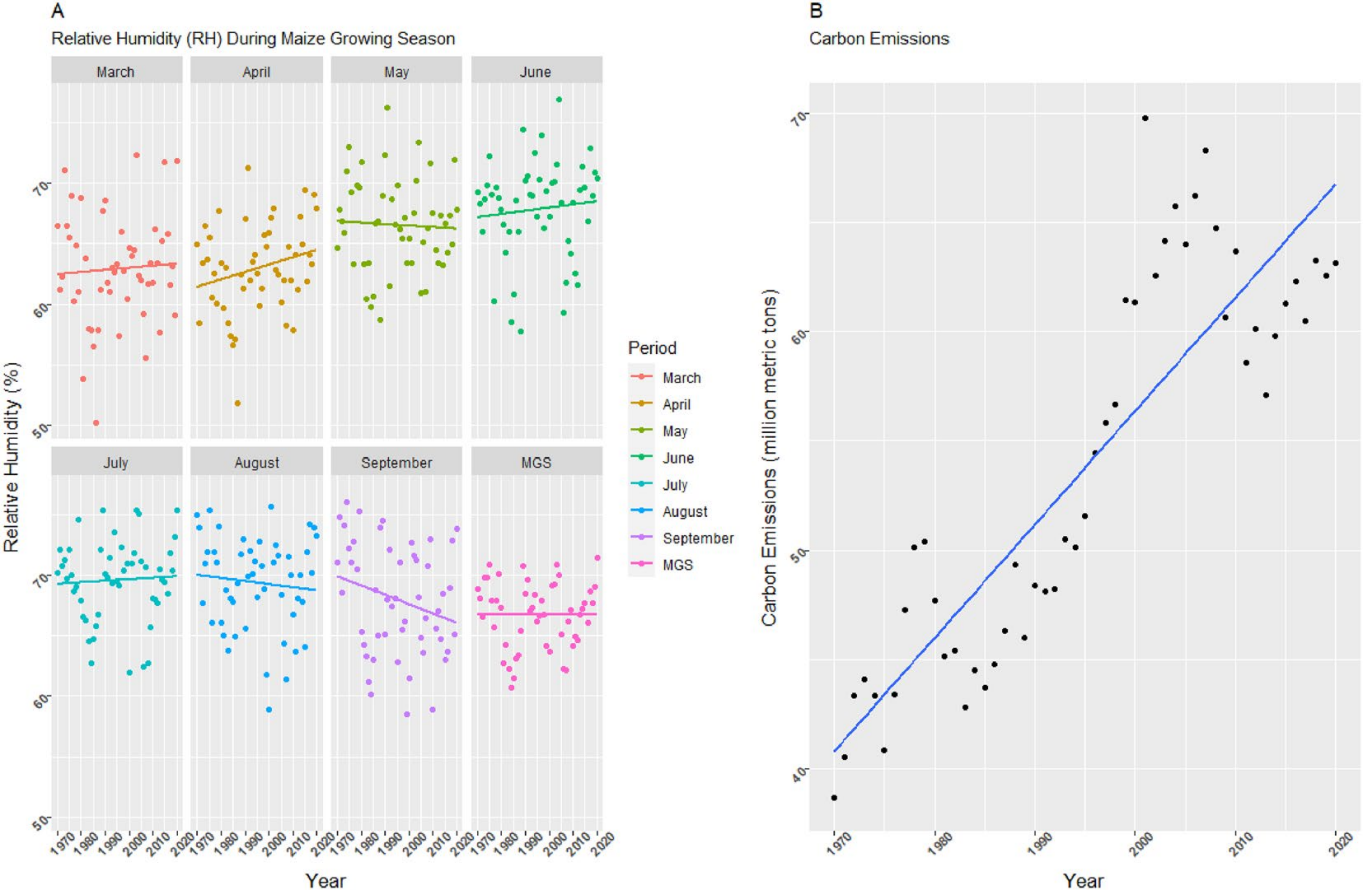
(**A**) Trend lines for relative humidity for maize growing season (MGS) and its individual months from 1970 to 2020 in Mississippi. (**B**)Trend line for CO_2_ emissions for years from 1970 to 2020 in Mississippi. Figure A is faceted by months from March to September and average of all months all together in MGS.

Precipitation and RH, neither for MGS nor for any other month were found to indicate a significant trend line (Figs. 5D, 6A), although numerically, a negative trend was noted in March, May, and September for PT and August and September for RH (Table 3).

A moderately strong and significant upward trend and an annual increase rate of 0.51 units was noted for CO_2_ (Fig. 6B; Table 3). The same is corroborated by Rahman^91^ and Wu et al.^92^ previously in the context of direction and strength, and by Ainsworth et al.^93^ in the context of rate of increase.

### The climatic impact on maize

The Tmax was found to have a significant negative effect on maize yield in both the short and long run (Table 4A). More specifically, every 1 °C rise in Tmax reduced the maize yield by 7.39% and 26.33% in the short and long run, respectively (Table 4A).

On further downscaling the analysis to monthly basis to capture the effect of within season variability, it was noted that the monthly averaged Tmax of June and July had a significantly negative correlation with maize yield (Table 4B). This indicates that Tmax in June and July (reproductive-early grain filling stages) contributed the most to yield loss in MS. This is because in reproductive stage, stress-induced plant dysfunction has irreparable harm on kernel development and yield which is not the case with the vegetative phase^94,95^. These findings are consistent with those of Kucharik and Serbin^17^ in the context of highly correlated months with respect to maize growing season and to those of Lobell and Field^23^, and Wu et al.^92^ in the context of Tmax's adverse effects. Hu and Buyanovsky^96^ reported that maize needs both a warming trend with temperatures higher than average in April and May to provide better conditions for germination and emergence and a cooling trend with temperatures lower than average in June-August to promote reproductive success and, consequently, yield. This statement is largely agreed with by Lobell and Asner^97^ as well. However, MS had not seen any significant warming trend in April and May; instead, it showed an unfavorable significant warming trend in June and August (Table 3). Contrary to favorable conditions, MS was observed to have temperatures that were below average (28.56 °C) in April (24.24 °C) and May (28.13 °C) and above average in June (31.66 °C) and August (32.78 °C) (Table 3). The Mid-MGS (*i.e.*, the beginning reproductive stage) coincides with June and July (hotter climate), which affects tasseling and grain filling, thereby yield, and is sensitive to additional warming^98,99^. Furthermore, the average Tmax (28.56 °C) noted in MS for MGS (Table 3) has already surpassed the optimal temperature (26.40 °C) for maize^100^, and is rapidly approaching 29 °C, which is damaging to maize^101^. The main reason is that after surpassing 29 °C^101,102^ or 30 °C^103^, processes such as anthesis-silking, assimilates production, translocation of resources during reproductive and grain filling are hampered. Temperature beyond this range has been linked to impaired pollen structure, decreased sugar (energy) levels upon anthesis, and retarded pollen shedding, all of which negatively affect pollen germination ability and fertilization^104^. More recent studies found that short duration of Tmax episodes during anthesis can cause significant reduction in pollen germination (30%), kernel number (72%), kernel weight (10%), and stomatal conductivity (52%) in maize^105,106^. Further at the biochemical level, the activity of the enzymes involved in converting atmospheric CO_2_ to glucose or other key photosynthesis-related molecules were found to be disrupted by elevated temperatures^107^. In worst case scenario at higher temperatures, a yield loss could reach 34–80%^87,108^.

A 1°C rise in Tmin increased maize productivity by 20.68% over the long run, indicating a significant and positive effect on maize yield in MS (Table 4A). Several other maize-growing regions have shown that yields respond to Tmin^87,109–111^. Tmin warming was also shown to be advantageous to maize yield in the short run, while the impact was not significant (Table 4A). Although there has not yet been an agreement regarding the physiological effects of Tmin on plants as there is an inclination of the crop-climate research towards the Tmax or Tavg and overlooking the Tmin^112,113^. The current study's findings on the positive association of Tmin and maize yield were supported by evidence from the literature, which included studies using statistical modeling^87,114–121^ as well as simulation-based studies^122,123^. This is attributable to the fact that the increased Tmin speeds up night-time respiration, resulting in carbohydrates losses^124^. However, this carbon starvation enhances the following day photosynthetic rate to more than make up for the losses brought on by the accelerated night-time respiration, increasing overall plant productivity^125,126^. Consequently, the amassed dry matter from various plant tissues starts remobilizing toward grain, increasing maize kernel weight, and hence, the yield^127^. Also, the increased Tmin is believed to impart conducive conditions for germination, emergence, seedling growth, grain filling (during night-time), and milk-maturity stage in maize^110^. More importantly, according to Badu-Apraku et al.^127^, Cairns et al.^128^, and Sanchez et al.^100^, all the beneficial mechanisms of Tmin mentioned above only prevail when the Tavg is below 25 °C or 26.40 °C. The Tavg for the current study was found to be 22.29 °C (Table 3). Furthermore, a similar case of Tavg of less than 25 °C was observed in all studies that supported the current findings, specifically at 21.2 °C and 24.4 °C in Liu et al.^116^ and Shammi and Meng^36^. Contrarily, the studies that found negative effects of Tmin on maize yield were all found to have been carried out at Tavg of more than 25 °C^129^. For example, Wang et al.^130^ tested at Tavg (27-31 °C), Liu et al.^131^ tested at Tavg (25–35 °C), Suwa et al.^132^ at Tavg (31 °C), and Wilhelm et al.^133^ at 29.5 °C and observed negative Tmin-yield impact in maize. Furthermore, it was noted that June, July, and August demonstrated a significant and positive correlation between Tmin and detrended yield (Table 4B). This suggests that warmer nights in June, July, and August are beneficial for maize yields in MS, but there is no evidence that this beneficial effect offsets the detrimental effect of Tmax during the same months. Chen et al.^110^ also noted 1 °C Tmin warming during May/September improved maize yield by 303/284 kg ha^-1^. Reilly^134^, Izaurralde et al.^135^, and Reilly et al.^136^ also realized the positive effects of warming on maize yield. Also, according to Schlenker and Roberts^137^, Lobell et al.^138^, and Lobell et al.^139^, yield reductions are expected when temperature surpasses 30 °C, which was not the case with this study (Table 3). So far, the curve of Tmin has never reached the point at which it can cause the Tavg to pass above the optimal range and negatively affect maize yield.

According to the model's long-run estimation, the rising trend in CO_2_ emissions had a positive and significant impact on maize yield (Table 4A). Ahsan et al.^140^ and Chandio et al.^40^ also realized similar yield improvements due to CO_2_ emissions. However, it was discovered that the impact of CO_2_ emissions on maize yield in the short run was not significant (Table 4A), and this is consistent with Warsame et al.^55^ and Anapalli et al.^38^ studies, focused on MS. Specifically, every unit increase in CO_2_ emissions resulted in a long-term improvement in maize yield of 0.62% (Table 4A). Similar reports of 0.23% and 0.70% yield increases were noted by Asfew and Bedemo^56^ and Mahrous^141^ where they quantified the positive effects of increased CO_2_ emissions. However, Islam et al.^142^ estimated that under current climate change scenarios, these CO_2_ emissions-driven yield increments might reach 3.5 to 12.8% at the rate of 1.80% every decade^143^. The upsides of elevated CO_2_ on maize yield are due to its effects on plant physiology, growth, and biochemistry, through diminished stomatal conductivity and enhanced photosynthetic rates^144–147^. The decreased stomatal conductance reduces water loss thereby increasing water use efficiency, especially in drought-stress conditions^148,149^. The rise in atmospheric CO_2_ levels increases the intercellular CO_2_ concentration (Ci) and thus, photosynthetic rate (A)^150^. However, maize has a lower carbon saturation point than C3 plants like soybean^151^ due to the high affinity (to CO_2_) of the key enzyme, phosphoenolpyruvate carboxylase^152,153^. These physiological and biochemical responses of maize to CO_2_ indicated that further increases in CO_2_ levels may not increase assimilation production^150,151^. Increased CO_2_ level have been shown to benefit other crops^154–157^. However, the response of C4 plants (maize) to elevated CO_2_ levels is complex, as it is influenced by various factors such as air temperature, water availability, light intensity, vapor pressures, and nitrogen availability^158,159^. Nevertheless, predicted rise in CO_2_ levels by the years 2050 and 2100 may diminish the beneficial effect of CO_2_ in row crops, like maize^150,151^. Further research is therefore required to determine the influence of elevated CO_2_ in C4 plants at different growth stages^150,152,160,161^

Even though PT is a crucial crop growth factor, the current findings revealed that, at a 1% level of significance, PT patterns were determined to pose a negative and significant effect on maize yields in both the short- and long-term (Table 4A). More specifically, every 1 mm change in PT had reduced maize yield in the short- and long-term, by 0.64% and 2.70%, respectively (Table 4A). These results are consistent with the observations of Rosenzweig et al.^162^, Chen et al.^163^, and Xiang and Solaymani^58^ who also noted the negative effect of the ongoing PT trends on maize yield. A crop yield decline due to prevailing PT trends was also documented in the study by Shammi and Meng^36^ in MS. These results are attributable to the excessive PT (1504.44 mm annually) in MS^164^. Excessive PT, in addition to directly or physically harming the crop, results in prolonged wet conditions that lead the soil saturation and are averse to crop development, particularly in conditions of inadequate drainage^165^. This yield-reducing effect of excess moisture is attributable to (i) root growth hindrance impairing plants ability of nutrients and water uptake^166,167^, (ii) increased nitrate leaching, leading to nutrient depletion^168^, (iii) anoxic conditions in soil, leading to the risk of toxic substances development, diseases, and insect infestation^169^, and (iv) delayed planting or harvesting, owing to the difficulty of driving the machinery in wet fields^149,170,171^. On account of the aforementioned factors, the US as a whole suffers a 3% yield loss annually^162,172^, and significant yield decline has been seen over the past two decades in various parts of the US *i.e.,* Iowa^173,174^. When the analysis was further scaled down to a monthly level, it was discovered that the most significant month correlated with the MS maize yield was August, and the association was negative (Table 4B). This indicates that the August PT had the most significant negative effect on MS maize, and Eck et al.^82^ also deduced similar results documenting increased PT to be detrimental in the latter part of the MGS. This is because the uptake of nitrogen, phosphorus, and potassium in maize plants continues up until the R3-R4 stage in August, when the plant can still transpire to the extent of 0.25–0.30 inches of water, according to Lauer^175^, who claimed that by this time, the two (ear and kernel number) of three key yield parameters are determined, but the kernel size/weight is still yet to be determined. Furthermore, low PT is required during the ripening period (August) of maize^96^; nonetheless, the current study found that the MGS month with the highest PT growth rate (2.69 mm/decade) was August (Table 3). However, Rosenzweig et al.^162^ had a different perspective on the negative association of August-maize yield, according to them it probably has less to do with plant itself and more primarily linked with the harvesting challenges arising from overly moist conditions, for growers. Delayed harvesting degrades the quality of maize, rendering it unsalvageable, in some instances, due to rotting in the field^82^. Overall, such scenarios of delayed harvesting could lead to a yield loss to the extent of 10%^149^.

Pearson’s correlation matrix revealed that the RH of any month of MGS had no correlation but DTR of June, July, and August months had negative and strong correlation with the maize yield (Table 4B). These results are consistent with those of Muhammad et al.^176^ who found a weak correlation of RH and HA with yields, as well as with that of Lobell^89^ who examined the impact of DTR on maize yield.

The coefficient of ECM was determined to be  − 0.302 (Table 4A), which signifies that every year, 30.20% of the immediate climatic impact cumulatively transfers to form the permanent basis for the long-term effects. A 30.20% is equivalent to the results of Warsame et al.^55^ and Jan et al.^44^. The ARDL model estimated the adjusted R^2^ value of 0.766, indicating that 76.60% of the total variations in maize yield due to the studied variables are explained by the study model.

### Study limitations

Each research has its unique set of limitations, which forms the base for further advancement in the research field. The factors such as maize evapotranspiration, sunshine durations/hours, irrigation intensity, and vapor pressure deficit that could interact to determine the climatic effects for better insights on crop-climate link, were not included in the present study due to data unavailability. Hence, future research is suggested incorporating the aforesaid variables along with the variables considered in the present study for more practicable and accurate estimations.

## Concluding remarks

This study demonstrated a markedly rising trend in Tmax, Tmin, and CO_2_, with Tmin majorly contributing to the overall warming trend in the MGS of MS. The Tmin progressed at a faster rate (0.14°C decade^-1^) than the Tmax, causing a considerably lowering trend in the DTR. The month-wise analysis determined the most correlated month for Tmax (June and July), Tmin and DTR (June, July, and August), and PT (August) in significantly impacting maize yield in MS, indicating the varied sensitivity of maize yield to within season variability for different climatic parameters. The crop-climate link assessment revealed a significantly negative effect of Tmax and PT on maize yield in both short and long run, whereas Tmin and CO_2_ emissions posed a significantly positive effect on maize yield in long run and no effect in short run. Overall, the study model explained the 76.60% variations in maize yield due to climate change in MS. As shown by the ECM coefficient of the study model, the short-term immediate climatic effects on maize progressively transfer to permanent long-term effects by 30.2% every year, making the crop-climate link more prominent in the long run than in the short run. As the water and nutrient usage efficiencies are climate driven and based on the current findings, it is suggested to reassess the agronomic optimum management strategies in the face of MS crop-climate link. Also, the research efforts need to be intensified to test crop varieties that might be more resistant to elevated Tmax, perform better under delayed planting circumstances, and continue to interact favorably with elevated CO_2_ and Tmin scenarios under the local climatic conditions of the MS. Moreover, it is recommended to test current findings at the field or in controlled settings using the locally prevalent climatic indices with a focus on agronomic optimum management strategies as they react to the climatic variations.

## Data Availability

The data used in this study is accessed from National Agricultural Statistics Service's repository (USDA-NASS), US Climate Divisional Database (NOAA), PRISM database, and US energy information administration. The online links for these data sources are mentioned in Section “Data” (data) of methodology chapter. However, for more information on data, rs2564@msstate.edu (Ramandeep Kumar Sharma) can be contacted. No separate field study on plants was carried out because all the data used in the study was accessible online.
